# Effects of Aβ exposure on long-term associative memory and its neuronal mechanisms in a defined neuronal network

**DOI:** 10.1038/srep10614

**Published:** 2015-05-29

**Authors:** Lenzie Ford, Michael Crossley, Thomas Williams, Julian R. Thorpe, Louise C. Serpell, György Kemenes

**Affiliations:** 1 Sussex Neuroscience, School of Life Sciences, University of Sussex, Brighton, BN1 9QG

## Abstract

Amyloid beta (Aβ) induced neuronal death has been linked to memory loss, perhaps the most devastating symptom of Alzheimer’s disease (AD). Although Aβ-induced impairment of synaptic or intrinsic plasticity is known to occur before any cell death, the links between these neurophysiological changes and the loss of specific types of behavioral memory are not fully understood. Here we used a behaviorally and physiologically tractable animal model to investigate Aβ-induced memory loss and electrophysiological changes in the absence of neuronal death in a defined network underlying associative memory. We found similar behavioral but different neurophysiological effects for Aβ 25-35 and Aβ 1-42 in the feeding circuitry of the snail *Lymnaea stagnalis.* Importantly, we also established that both the behavioral and neuronal effects were dependent upon the animals having been classically conditioned prior to treatment, since Aβ application before training caused neither memory impairment nor underlying neuronal changes over a comparable period of time following treatment.

Alzheimer’s disease (AD) is the most prevalent neurodegenerative disorder in the Western world, affecting an estimated 24.3 million individuals globally. AD is characterized by progressive memory loss and the prominent markers are extracellular amyloid plaques, made of amyloid β (Aβ), and intracellular neurofibrillary tangles, composed of hyperphosphorylated tau^1^. Aβ are secreted peptides that have been cleaved from the amyloid precursor protein (APP)^2^, with the oligomeric form of the peptide being widely regarded as the toxic structure^3^. The role of Aβ as a toxic element in the disease has been widely explored through multiple techniques, including *in vivo* and *in vitro* application of synthetic peptides as well as in novel transgenic mouse models created to overexpress Aβ. Transgenic models, such as Tg2576, have shown a correlation between increased Aβ production in the brain and decreased performance in numerous memory tasks^4^. Many laboratories have studied the effect of Aβ using varying fragments of the Aβ peptide, model system, and concentration applied, collectively agreeing that Aβ interferes with long term potentiation (LTP) and early memory^5^,^6^,^7^. However, contrasting results exist between different transgenic mouse lines, types of memory being tested, and areas of the brain being observed (for review, see^8^). These contradictions are widespread in the Aβ field. A common problem is that experiments are often performed on animals, cells, or tissues that are already exhibiting neurodegenerative properties, potentially masking any early-stage symptoms of Aβ treatment. Death of the neurons that encode a memory trace will inevitably be manifested as a behavioral deficit. However, synaptic dysfunction and impairment of intrinsic plasticity have been found to occur before neuronal cell death in Aβ models, causing sufficient disturbance to result in learning and memory dysfunction^3^,^9^,^10^,^11^,^12^. We believe that these early-stage disruptions are excellent targets for drug and clinical interference with AD and that more emphasis needs to be placed on pre-cell death studies.

For this reason, we have examined the effects of Aβ treatment on the maintenance of established long-term memory (LTM) and underlying neuronal properties in the pond snail, *Lymnaea stagnalis,* with no signs of cell death in the neuronal circuitry for feeding that both acquires and stores the implicit memory trace after food-reward classical conditioning^13^. Invertebrates are used for their simplicity and ability to allow techniques to be used that would be extremely difficult, if not impossible, to accomplish in a mammalian model. *Lymnaea* provides a highly tractable model system that is well established in the learning and memory field for its uses in behavioral, electrophysiological, molecular, and cellular network experimentation, allowing for both *in vivo* and *in vitro* approaches^14^. The use of *Lymnaea* allows a top-down approach to be taken, with multiple techniques being used on the same individual animals and same specific neurons. This allows for a direct link to be established between peptide administration and behavioral and neuronal changes, a crucial component to Aβ research that is currently lacking in other models. Previous work in a similar snail model has found that *Helix lucorum* were unable to learn a conditioned food aversion reflex when repeatedly injected with high concentrations of Aβ 25-35, 24 hours before and 72 hours after training, in conjunction with a 5-day multi-trial training protocol. Moreover, facilitation of synaptic responses was significantly reduced when the central nervous system was exposed to Aβ 25-35^15^. However, the effect of Aβ treatment on consolidated memory was not investigated in *Helix* and has been a relatively unexplored form of memory throughout the literature. Consolidated memory was successfully investigated by Lesne *et al.* 2006^16^, where they administered Aβ dodecamers into rat brains two weeks post-training, allowing the group to conclude that Aβ disrupted consolidated memory but not memory acquisition.

Here, we used a pre-cell death Aβ incubation time point combined with a single-trial food-reward classical conditioning paradigm to investigate the effects of Aβ on associative LTM in *Lymnaea*. We found that snails treated with either a short amyloidogenic fragment (Aβ 25-35) or full-length Aβ 1-42 had significantly impaired memory 24 hours after treatment. We also found that memory loss associated with administration of Aβ 25-35 was correlated with impaired non-synaptic plasticity in key neurons within the memory and feeding network, whilst Aβ 1-42 affected memory through impairing a different, possibly synaptic, mechanism. Importantly, Aβ induced changes in behavior and neuronal properties only occurred when training preceded injection, suggesting a detrimental effect on memory maintenance or late/lingering consolidation rather than acquisition.

## Results

### Systemically applied Aβ 1-42 enters the snail brain

**Figure 1:**
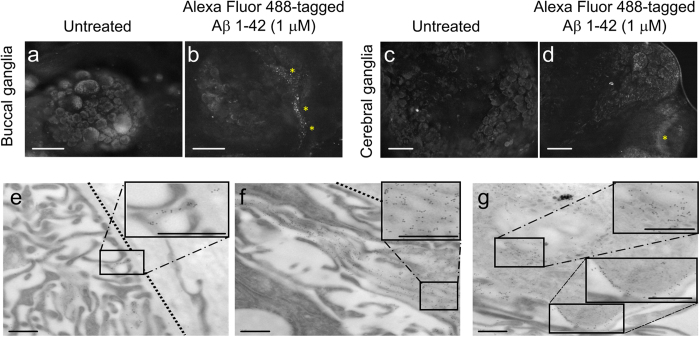
AlexaFluor 488-tagged Aβ 1-42 (1 μM) reaches and penetrates the snail brain within 24 hours of *in vivo* incubation. (**a–d**) Wholemount fluorescent images of 1 μM AlexaFluor-Aβ, or vehicle treated brains after 24 hour *in vivo* incubation. Representative fluorescent images of untreated (**a** and **c)** and Aβ treated (**b** and **d)** desheathed buccal and cerebral ganglia. Yellow asterisks indicate the likely presence of AlexaFluor-Aβ. Scale bars represent 100 μm. (**e–g)** Immunogold labeled transmission electron micrographs of 1 μM AlexaFluor-Aβ, 24 hour *in vivo* incubation cerebral ganglia sections. 10 nm gold labels indicate the presence of Alexafluor**-**Aβ outside of the ganglia **(e)** as well as inside the cellular projections near the ganglion edge (**f)** There is also an accumulation of gold labeled Aβ within cellular projections, well within the ganglia (**g)** Gold labels within cells indicate that Aβ enters cells (top insert in **g**) but Aβ also localizes outside of the cell membrane (bottom insert in **g**). Scale bars represent 0.5 μm.

One benefit of using *Lymnaea stagnalis* in behavioral pharmacological studies is that the animal has no blood-brain barrier^17^; therefore, it is not necessary to directly apply Aβ to the brain tissue and the Aβ concentration can be kept low and well controlled for each individual animal. We used this to our advantage and monitored the entry of systemically injected Aβ into the buccal and cerebral ganglia, which contain all the circuitry involved in memory after food-reward conditioning^13^. An Alexa Fluor 488 Protein Labeling Kit was used to tag freshly prepared Aβ 1-42 (see Methods). The peptide was measured with respect to fluorescence and degree of labeling. The freshly prepared Alexa Fluor 488- tagged Aβ was immediately injected into the animals at the working concentration of 1 μM and allowed to incubate *in vivo* for 24 hours. Whole brains were dissected and desheathed of the perineurium, the protective connective tissue, and viewed using a fluorescence microscope. While non-specific tissue autofluorescence does exist in snail neurons, possibly masking some of the fluorescence of the 1 μM Aβ 1-42, samples from animals treated with the tagged peptide exhibited distinct fluorescent punctae indicating the presence of Aβ 1-42 (Fig. 1a–d).

The areas with the highest degree of labeling appeared to be where perineurium remained around the ganglia, with some possible labeling inside the ganglionic tissue. Although these results indicated that Aβ 1-42 reaches the snail brain within 24 hours after systemic injection, we were unable to establish if Aβ 1-42 actually penetrated the ganglia and neurons using light microscopy alone. Therefore, we used the same Alexa Fluor 488-tagged Aβ 1-42 treated ganglia and viewed immunogold labeled sections under the transmission electron microscope (TEM). Using a primary antibody specific for the Alexa Fluor 488 tag and a 10 nm gold-tagged secondary antibody, we were able to visualize the location of Aβ 1-42 at the ultrastructural level. Figure 1e–g reveals Alexa Fluor 488-tagged Aβ within the ganglia and these peptides can be tracked throughout the brain tissue and inside cells. Upon determining that Aβ 1-42 does enter ganglia and cells, we focused on organelles with a high degree of labeling. Aβ 1-42 labeling was found within the nucleus, mitochondria, and dense-core granules/vesicles (Supplementary Fig. 1). There were also areas of highly labeled perineurium (Supplementary Fig. 1), suggesting that much of the systemically applied Aβ 1-42 gets caught in the connective tissue surrounding the brain.

### Oligomeric Aβ is present in the hemolymph 24 hours after injection

**Figure 2:**
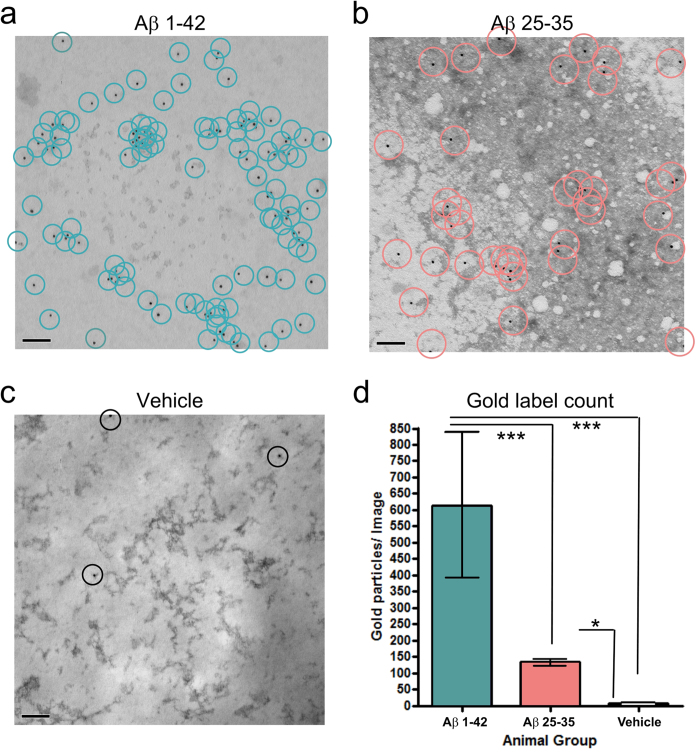
Oligomeric Aβ is found in the hemolymph after 24 hour *in vivo* incubation. **(a–c)** Micrographs of negative stained and Nu1 immunogold labeled, formic acid extracted hemolymph from animals treated with either 1 μM Aβ 1-42 (**a**) 0.1 mM Aβ 25-35 (**b**) or vehicle (**c**) after 24 hour *in vivo* incubation. Circles represent immunogold labels counted. Scale bars represent 100 nm. (**d**) Quantitative comparison of immunogold labels present in x20k magnification micrographs. Aβ 1-42 n = 4, Aβ 25-35 n = 24, Vehicle n = 13. Means ± SEM values are shown. Asterisks indicate significant differences in the number of gold particles per image between groups. One-way ANOVA, p < 0.0001. Tukey’s tests with p < 0.001: Aβ 1-42 vs Aβ 25-35 and Aβ 1-42 vs Vehicle. Tukey’s tests with p < 0.05: Aβ 25-35 vs Vehicle.

As the oligomeric forms of Aβ peptides are widely regarded as the toxic structure^3^, we conducted an experiment in which we administered either 1 μM Aβ 1-42, 0.1 mM Aβ 25-35 (based on the use of this concentration in *Helix*^15^, also see below), or vehicle, and incubated the animals for 24 hours before their hemolymph (body fluid), was extracted and subjected to formic acid extraction to select for soluble Aβ (see Methods). The hemolymph extracts were added to TEM grids, negative stained, and labeled with the oligomer-specific Nu1 primary antibody^18^ and a 10 nm gold-conjugated secondary antibody. Immunogold particles were observed in large numbers in both peptide-treated hemolymph samples (Fig. 2a,b), but were negligibly detected in vehicle-treated controls (Fig. 2c). Notably, Aβ 1-42 has significantly more Nu1- labeling than Aβ 25-35 regardless of the 100-fold higher injected concentration of Aβ 25-35 (Fig. 2d). The presence of these immunogold particles in the hemolymph extracts indicates that even at 24 hours after systemic administration, soluble and oligomeric Aβ 1-42 and Aβ 25-35 are still available for uptake by the nervous system and that Aβ 1-42 remains in a more oligomeric form than Aβ 25-35 after 24 hours *in vivo*.

### Both Aβ 25-35 and Aβ 1-42 disrupt consolidated long-term memory

**Figure 3:**
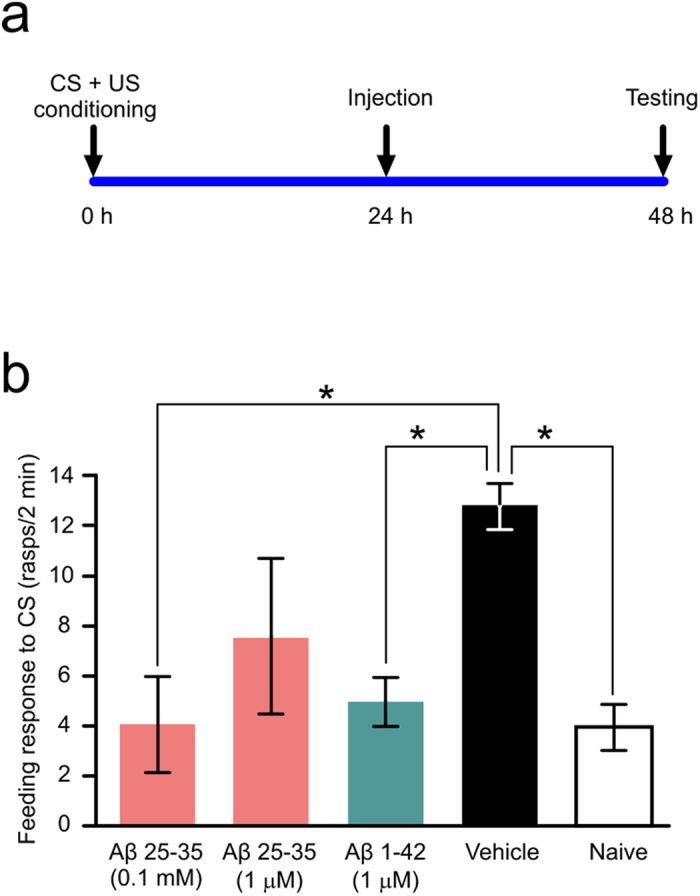
Aβ treatment leads to memory loss. (**a**) Time line of the experiment. (**b**) Five starved animal groups (0.1 mM Aβ 25-35, n = 18; 1 μM Aβ 25-35, n = 24; 1 μM Aβ 1-42, n = 55; vehicle, n = 96; naïve, n = 55) were tested for rasp rate to amyl acetate, a measure of the feeding response to the CS. Means ± SEM values are shown. Asterisks indicate behavioral responses that are significantly lower than those in the vehicle treated group. One-way ANOVA, p = 0.0001. Tukey’s tests with p < 0.05: Vehicle vs. Naïve, Aβ 1-42 vs. Vehicle, 0.1 mM Aβ 25-35 vs. Vehicle.

The short fragment peptide Aβ 25-35 has been shown to have neurotoxic properties and to affect cognitive processes^19^, and has been used successfully in another molluscan model^15^. The fragment represents the core functional domain of the full length Aβ peptide and is able to self-assemble to form a predominantly β-sheet structure. For this reason, it has been used to test the effects of Aβ exposure^19^. Therefore, our initial experiments to test the effect of Aβ on behavior were conducted with Aβ 25-35. Animals were classically conditioned using a food-reward training paradigm (Fig. 3a). Treatment with 0.1 mM Aβ 25-35 at 24 hours after training significantly reduced the animals’ feeding response to the conditioned stimulus (CS) tested 24 hours after injection and 48 hours after training (Fig. 3b). This concentration was investigated due to its demonstrated effect on memory in *Helix*^15^. However, 0.1 mM Aβ 25-35 is considered to be a rather high concentration, so we administered a more commonly used concentration of 1 μM Aβ 25-35 for comparison. The data showed a trend for decreased memory but no significant memory impairment (Fig. 3b), therefore all subsequent experiments were conducted using 0.1 mM Aβ 25-35.

While Aβ 25-35 (racemized at D-Ser^26^) has been immunohistochemically detected in plaques of AD brains^20^, the peptide is not actively cleaved from the APP protein. Therefore, the effect of Aβ 1-42, the most toxic of the Aβ peptides with a widely accepted physiological relevance, was also examined. At 1 μM, Aβ 1-42 significantly reduced the animals’ feeding response to the CS (Fig. 3b). As a control, trained animals were injected with vehicle and no memory impairment was observed. Therefore, the behavioral effect observed is not a result of either the buffer the Aβ is solubilized in or the injection itself. Both 1 μM Aβ 1-42 and 0.1 mM Aβ 25-35 decreased the animals’ response rates to baseline, naïve levels. Importantly, neither peptide affected the unconditioned feeding response to sucrose when tested 24 hours after systemic injection (Supplementary Fig. 2a). The lack of feeding response to the CS therefore was not due to an impairment of the animals’ ability to generate the feeding motor pattern, which also indicates that the circuitry, within which the memory is encoded, is functioning healthily.

### Aβ 1-42 and Aβ 25-35 do not cause neuronal death or network dysfunction after 24 hours *in vivo* incubation

The most obvious cause of Aβ’s detrimental effect on memory would be the induction of cell death in the memory-encoding circuitry. We intended to observe pre-neuronal death time points in these experiments and so used four different indicators to monitor possible apoptosis or necrosis.

First, we tested the unconditioned feeding response to sucrose to monitor circuitry function through behavioral methodology. If Aβ-induced neuronal death was occurring within the memory and feeding network, the animals would not have a response rate to the unconditioned stimulus (US) as high as the uninjected, naive group since both the conditioned and unconditioned feeding motor program are controlled by the same neuronal circuit^13^. However, there was no statistical difference among the unconditioned feeding response rates of the 0.1 mM Aβ 25-35, 1 μM Aβ 25-35, 1 μM Aβ 1-42 injected and untreated animals (Supplementary Fig. 2a) already indicating that the feeding network was functioning properly.

Second, we used TEM to examine ganglionic sections from treated animals for evidence of apoptotic morphology within neurons. No evidence of apoptosis was observed in 1 μM Aβ 1-42 or 0.1mM Aβ 25-35 treated animals when compared to vehicle-injected animals and scored for health of the nucleolus, nuclear envelope, chromatin, and cell membrane (Supplementary Fig. 2b–h).

**Figure 4:**
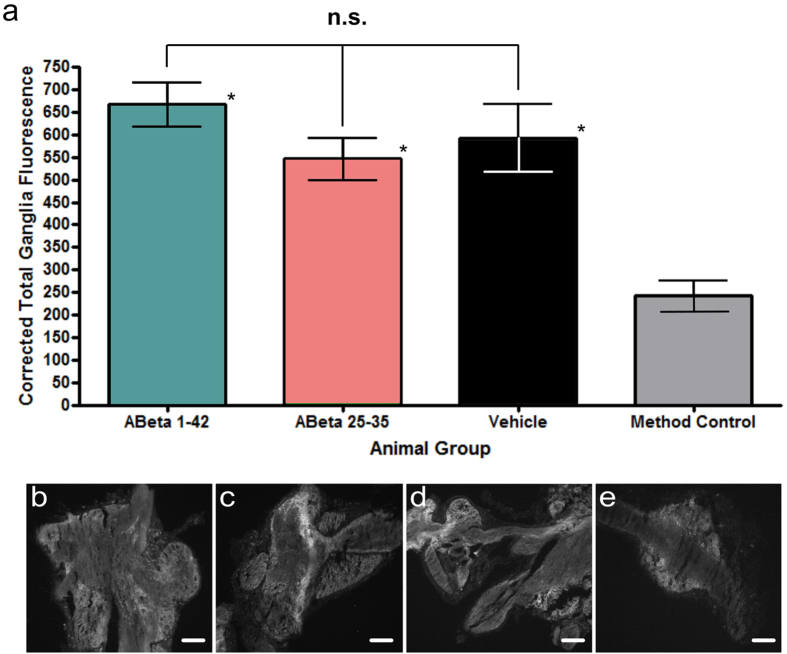
Aβ 25-35 and Aβ 1-42 do not cause apoptosis after 24 hour incubation. I. Annexin V assay. (**a**) Graphical representation of the amount of Annexin V, measured using an Alexa Fluor 488 signal. Ganglionic sections from four animal groups were imaged (Aβ 1-42, n = 28; Aβ 25-35, n = 18; Vehicle, n = 22; unlabeled naïve (method control), n = 10). Means ± SEM values are shown. Although signal intensity in each of the three experimental groups is significantly higher (asterisks) than that in the method control group, which only shows background autofluorescence (One-way ANOVA, p = 0.0003. Tukey’s tests, p < 0.05), the signal intensity is not significantly different (n.s.) among the experimental groups (Tukey’s tests, p > 0.05). (**b**) Representative fluorescence image of an Aβ 1-42 treated ganglionic tissue, labeled with Annexin V- Alexa Fluor 488. (**c**) Representative fluorescence image of an Aβ 25-35 treated ganglionic tissue, labeled with Annexin V- Alexa Fluor 488. (**d**) Representative fluorescence image of a vehicle treated ganglionic tissue, labeled with Annexin V- Alexa Fluor 488. (**e**) Representative fluorescence image of ganglionic tissue from naive animals, with no Annexin V- Alexa Fluor 488 added. Scale bars represent 100 μm.

Third, we used an Annexin V-Alexa Fluor 488 label on ganglionic sections from either 0.1 mM Aβ 25-35, 1 μM Aβ 1-42 injected, or vehicle injected animals and imaged them using fluorescence microscopy. These images were compared to sections with no Annexin V-Alexa Fluor 488 label as a method control. Annexin V will bind to phosphatidylserine (PS) on the cellular membrane when cells undergo apoptosis, so higher signal intensity in sections from the Aβ treated animals compared to vehicle controls would indicate Aβ-induced apoptosis in the snail brain. However, fluorescence signal intensity did not differ statistically among 0.1 mM Aβ 25-35, 1 μM Aβ 1-42 and vehicle injected animals (Fig. 4a, examples in Fig. 4b–d), indicating that there is no increased apoptosis in Aβ treated animals compared to the vehicle control. The significantly lower level of fluorescence in the method control (Fig. 4a, example in Fig. 4e) suggests that only about 30 to 40% of the fluorescence seen in the experimental groups is non-specific background fluorescence, likely due to the well-known autofluorescence of the snail ganglionic tissue. The rest of the fluorescent signal seen in the Aβ and vehicle treated groups is most likely due to the baseline Annexin V level found naturally in cells.

**Figure 5:**
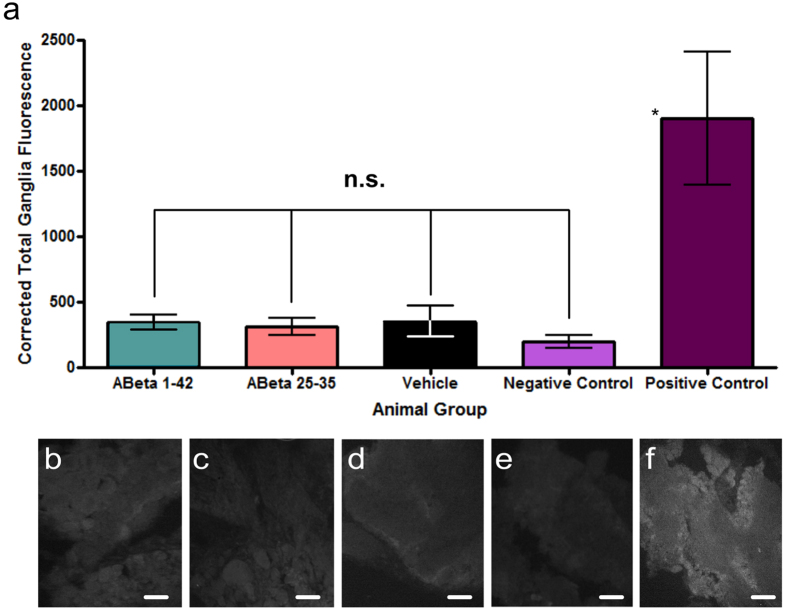
Aβ 25-35 and Aβ 1-42 do not cause apoptosis after 24 hour incubation. II. TUNEL assay. (**a**) Graphical representation of the amount of TUNEL signal in ganglionic sections from five animal groups (Aβ 1-42, n = 12; Aβ 25-35, n = 7; Vehicle, n = 7; negative control, n = 6; positive control, n = 3). Means ± SEM values are shown. The asterisk indicates significantly higher signal intensity in the positive control group compared against each of the other groups (One-way ANOVA, p < 0.0001. Tukey’s tests, p < 0.05). The signal intensity is not significantly different (n.s.) among the three experimental and the negative control group (Tukey’s tests, p > 0.05). (**b–f**) Representative fluorescence images of ganglionic sections in the three experimental and two control groups. (**b**) Aβ 1-42 treated, labeled with TUNEL, (**c**) Aβ 25-35 treated, labeled with TUNEL, (**d**) Vehicle treated, labeled with TUNEL, (**e**) Negative control, labeled with Labeling Solution only, (**f**) Positive control, labeled with TUNEL. Scale bars represent 50 μm.

Finally, we used a TUNEL assay on ganglionic sections from either 0.1 mM Aβ 25-35, 1 μM Aβ 1-42 injected, or vehicle injected animals and again imaged them using fluorescence microscopy. These images were compared to sections with no TUNEL reagent applied as a negative control and to ganglionic sections treated with DNase I as a positive control. TUNEL will bind the terminal end of nucleic acids after DNA fragmentation, an indicator of cell death, so a high signal would indicate a high level of apoptosis. However, TUNEL signal intensity did not differ significantly among the 0.1 mM Aβ 25-35, 1 μM Aβ 1-42 and vehicle injected experimental groups (Fig. 5a, examples in Fig. 5b–d) or among the experimental groups and the negative control group (Fig. 5a, examples in Fig. 5b–e). All three experimental groups, as well as the negative control, showed significantly less signal than the positive control (Fig. 5a, examples in Fig. 5b–f).

The lack of behavioral, morphological, protein and DNA indicators of cell death, for both the 1 μM Aβ 1-42 and the 0.1 mM Aβ 25-35, along with the memory impairment observed with both peptides in the previous experiment (Fig. 3b), justified the use of these concentrations to investigate pre-cell death effects of Aβ 1-42 and Aβ 25-35 on identified neurons of the learning and memory circuit.

### 24 hours *in vivo* incubation with Aβ alters neuronal properties

**Figure 6:**
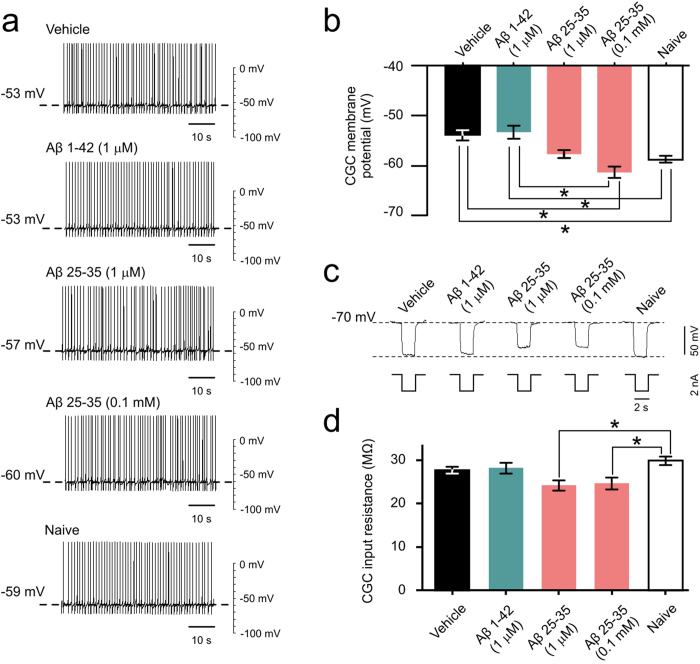
Electrophysiological effects of Aβ treatment. (**a**) Examples of electrophysiological recordings of CGC membrane potential and tonic firing activity under different treatment conditions. (**b**) Membrane potential data, represented graphically (1 μM Aβ 1-42, n = 12; 1 μM Aβ 25-35, n = 12; 0.1 mM Aβ 25-35, n = 15; vehicle, n = 26; naïve, n = 26). Means ± SEM values are shown. Asterisks indicate significantly weaker membrane depolarization in comparison to the vehicle-treated group. One-way ANOVA, p < 0.0001. Tukey’s tests with p < 0.05: 1 μM Aβ 1-42 vs. 0.1 mM Aβ 25-35, Vehicle vs. Naïve, Vehicle vs 0.1 mM Aβ 25-35. (**c**) Examples of electrophysiological recordings of CGC membrane resistance under different treatment conditions. (**d**) Membrane resistance data, represented graphically (1 μM Aβ 1-42, n = 12; 1 μM Aβ 25-35, n = 11; 0.1 mM Aβ 25-35, n = 15); vehicle, n = 24; naïve, n = 25). Means ± SEM values are shown. Asterisks indicate data that are significantly different than the vehicle-treated group. One-way ANOVA, p < 0.01. Tukey’s tests with p < 0.05: 1 μM Aβ 25-35 vs Naïve and 0.1 mM Aβ 25-35 vs Naïve.

The finding that both Aβ 25-35 and Aβ 1-42 impair established LTM without causing cell death led us to hypothesize that one or both of these peptides alters the electrical properties of key neurons known to be involved in the maintenance of LTM in *Lymnaea*. One such identified neuron type is the paired Cerebral Giant Cells (CGCs) that show a long lasting membrane potential depolarization after single-trial food-reward classical conditioning^21^ (Supplementary Fig. 3a,b). This depolarization emerges over a period of between 18 and 24 hours post-training, so it is involved in memory maintenance or late/lingering consolidation rather than acquisition or early consolidation^21^. Depolarization of the CGCs was found to be sufficient for the feeding response to be evoked by the CS after 24 hours post-training^21^,^22^. In a set of electrophysiological experiments, we therefore investigated if Aβ treatment of intact animals at 24 hours post-training affects a number of key electrical properties of the CGCs, including membrane potential, spike shape, and membrane resistance. Comparisons were also made with CGCs in preparations from control animals that were either naïve, or trained and injected with vehicle at the same time when the other three groups were treated with 0.1 mM Aβ 25-35, 1 μM Aβ 25-35, or 1 μM Aβ 1-42. Explicitly unpaired training does not cause electrical changes in the CGCs^21^ and so the use of this type of control in the present experiments was not necessary. Previous work has established that single-trial classical food-reward conditioning results in the delayed persistent depolarization of the CGCs by between 3 and 10 mV^21^,^22^. This result was replicated in our experiments (Fig. 6a,b), where the CGC membrane potential in the conditioned, vehicle-injected group of animals was more depolarized (by 5 mV on average) compared with preparations from naive animals. In accordance with previous findings^21^, classical conditioning did not result in significant changes in somal spike frequency, amplitude, peak, after-hyperpolarization, and half-width. These key parameters of the CGC action potentials were also not affected by treatment with the Aβ peptides (Supplementary Fig. 4), providing further confirmation of the health of the soma and the spike generating zones of the axon of these important neurons of the learning and memory circuitry.

Importantly, we found that Aβ 25-35 and Aβ 1-42 differed in their effects on the CGC membrane potential after classical conditioning. 0.1 mM Aβ 25-35 repolarized the membrane potential from a trained level to the level measured in naïve animals (by about 5 mV on average) while Aβ 1-42 had no effect (Fig. 6a,b). Similar to the behavioral results, the CGC membrane potential of the 1 μM Aβ 25-35 treated group was somewhere in between those of the 0.1 mM Aβ 25-35 and 1 μM Aβ 1-42 groups (Fig. 6a,b). However, both concentrations of Aβ 25-35 caused a significant reduction of the CGC’s membrane resistance compared to naïve animals whereas there was no significant difference among the other groups (Fig. 6c,d). These findings suggest that 0.1 mM Aβ 25-35 at least partially causes the observed memory loss through altering membrane properties of key neurons in the network, while Aβ 1-42 is altering memory through different, possibly synaptic, mechanisms.

The differences between the effects of the two peptides is possibly due to structural differences; therefore the morphology of Aβ 1-42 and Aβ 25-35 were examined over an assembly time of 24 hours *in vitro*. Electron micrographs showed that Aβ 25-35 forms small crystalline structures that develop into larger elongated crystalline structures after 24 hours. In contrast, Aβ 1-42 formed small, spherical oligomeric species that developed into protofibrils and then mature fibrils after 24 hours (Supplementary Fig. 5). The difference in morphology may account for the observed differences in cellular interactions.

### Training is required to induce behavioral and neuronal vulnerability to Aβ

**Figure 7:**
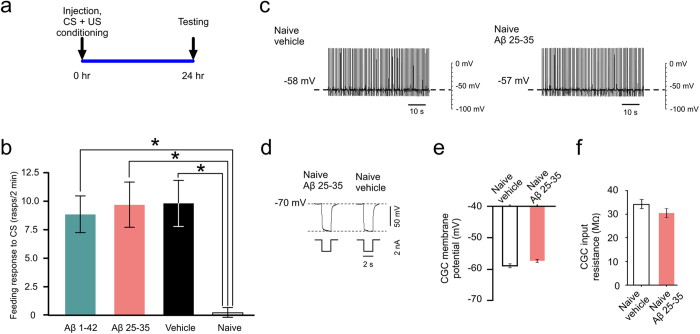
Aβ 1-42 and Aβ 25-35 exert their effects only after classical conditioning. (**a**) Time line of the experiment. (**b**) Four starved animal groups (Aβ 1-42, n = 27; Aβ 25-35, n = 30; vehicle, n = 16; naïve, n = 16) were tested for the feeding response to the CS 24 hours after injection and training. Means ± SEM values are shown. Asterisks indicate responses that are significantly higher than those in the naive group. One-way ANOVA, p = 0.0039. Tukey’s tests with p < 0.05: Vehicle vs. Naïve, Aβ 1-42 vs. Naïve, Aβ 25-35 vs. Naïve. (**c**) Examples of electrophysiological recordings of CGC membrane potential and tonic firing activity under different treatment conditions. (**d**) Examples of electrophysiological recordings of CGC membrane resistance under different treatment conditions. (**e**) The membrane potential data, represented graphically (Aβ 25-35, n = 9; vehicle, n = 5). Means ± SEM values are shown. t-test, p = 0.11. (**f**) The membrane resistance data, represented graphically (Aβ 25-35, n = 9; vehicle, n = 5). Means ± SEM values are shown. t-test, p = 0.26.

Previous studies have found that memory is impaired following multiple applications of Aβ^22^,^23^ or in a transgenic animal that over-produces Aβ^6^,^24^. However, these impairments arise from prolonged exposure to significant amounts of Aβ. Other studies have also looked at single injections of Aβ, but paired with multiple training trials^25^,^26^,^27^. This alters the time point being viewed and thus makes the ability to distinguish between memory acquisition and other stages, such as late stage consolidation, very difficult if not impossible. Our single-trial conditioning and pre-training single Aβ injection paradigm showed that treatment does not hinder memory acquisition at 1 μM Aβ 1-42 or 0.1 mM Aβ 25-35 concentrations when these peptides were applied immediately before training and allowed to incubate 24 hours *in vivo* (Fig. 7a,b). The effect of Aβ 25-35 applied at 1 μM concentration was not investigated due to the lack of behavioral response and lack of CGC membrane potential repolarization we found in the 24 hour post-injection tests, 48 hours after classical conditioning. Similar to the vehicle group, both Aβ 1-42 and Aβ 25-35 treated animals produced a significantly increased feeding response to the CS in comparison to naive animals.

We extended this study to analyze the effect of 1 μM Aβ 1-42 or 0.1 mM Aβ 25-35 injections applied immediately before training and allowed to incubate 48 hours *in vivo* (Supplementary Fig. 6a). By 48 hours post-training and post-injection, conditioned feeding response levels after both Aβ 1-42 and Aβ 25-35 treatment are significantly decreased, back to naïve levels, when compared to vehicle-injected animals (Supplementary Fig. 6b). These two experiments together brought to light the importance of the duration of the incubation with Aβ relative to memory phases. In *Lymnaea*, LTM is fully consolidated at 24 hours post-training and conditioned responses can be maintained for up to 14 days post-training^14^, so the difference in the effects of Aβ injection before training on 24 hour versus 48 hour memory is not due to testing different forms of memory at these two different time points. Instead, the difference observed here is likely due to the amount of time that Aβ is allowed to incubate *in vivo*. As we did not test for neuronal death after 48 hours of incubation, we cannot rule out that this had occurred by this later post-injection time point in the feeding and memory network. Although we show that Aβ does not affect the acquisition of 24-hour memory, it is possible that if the peptide is allowed to incubate long enough to disrupt the network, appropriate memory retrieval is not possible. However, the most parsimonious explanation for the deleterious effects of both Aβ peptides on memory at 48 hours is that at 24 hours post-training and post-injection there are still sufficient amounts of oligomeric peptides available to interfere with the consolidated memory trace, as was also indicated by our measurements of oligomeric peptide levels in the hemolymph (see Fig. 2).

We further tested the idea of training-induced susceptibility of neurons to Aβ-induced electrophysiological changes by testing CGCs in preparations made from naive, vehicle-injected animals and naive, Aβ 25-35-injected animals 24 hours after injection. This experiment established that in the absence of training, Aβ 25-35 did not cause membrane repolarization or decrease membrane resistance (Fig. 7c–f). These electrophysiological results, along with the behavioral data, suggest that training must precede Aβ 25-35 injection in order to observe any change in membrane properties and that training must precede injection in order to observe a behavioral change after 24 hours of incubation with either peptide. This suggests that either Aβ has no effect on memory acquisition or needs training to precede peptide exposure to disrupt the system.

## Discussion

We have identified a direct link between administration of Aβ and loss of consolidated LTM in *Lymnaea*, a well-established and highly tractable model system for studying evolutionarily conserved cellular and molecular mechanisms of memory function and dysfunction^14^. Importantly, our study revealed that systemically applied Aβ is able to enter the neurons of the ‘learning ganglia’ and leads to memory impairment at the behavioral level without any evidence for neuronal death. Both 1 μM Aβ 1-42 and 0.1 mM Aβ 25-35 caused significant memory impairment when injected 24 hours after training; however, only 0.1 mM Aβ 25-35 removed the training-induced membrane potential depolarization and lowered membrane resistance of a key neuron of the learning and memory circuitry. This is an important finding, as currently the two peptides are often used interchangeably throughout the literature. Although the peptides both caused similar behavioral dysfunction in *Lymnaea*, they affected the same identified neuron type in drastically different manners. Notably, we have demonstrated that 24 hours after injection, oligomeric forms of both Aβ 1-42 and Aβ 25-35 are present in the hemolymph. These toxic structures^3^,^5^,^28^ may be responsible for causing both the behavioral deficits and altered biophysical properties reported here. The morphological differences observed by TEM *in vitro* of Aβ 1-42 and Aβ 25-35 may explain the differences in their effects on the biophysical properties of key neurons of the learning and memory network, providing alternative pathways to the behavioral deficits observed. It appears that the larger crystalline structures formed by Aβ 25-35 lead to the reversal of learning-induced membrane depolarization, which is sufficient for the memory deficits observed. In contrast, the Aβ 1-42 peptide forms oligomeric species that lead to memory loss via alternative mechanisms, likely synaptic in nature. While the health of the CGC was confirmed in our electrophysiological studies based on recording from the cell body, future investigations will need to address the health of the synapses, which have been shown to be targets for Aβ and the first parts of neurons to become dysfunctional^3^,^9^,^12^.

**Figure 8:**
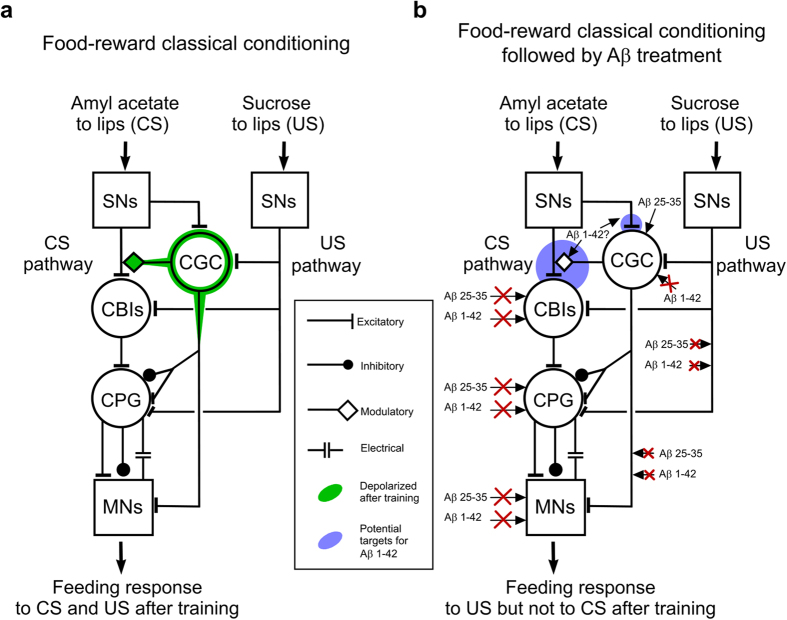
The proposed cellular and circuit mechanisms of Aβ-induced loss of memory retention in *Lymnaea*. (**a**) Twenty-four hours after training, the membrane potential of the CGC soma and proximal axonal segments are persistently depolarized (indicated by green outlines) allowing the gating in of CS-evoked sensory input to the CBI cells (Cerebral Buccal Interneurons). The CBIs in turn activate interneurons of the feeding central pattern generator (CPG) to generate the rhythmic motor pattern underlying the conditioned feeding response to the CS. Unconditioned food stimuli (such as sucrose) activate feeding via a parallel pathway not affected by classical conditioning. MNs, motoneurons of the feeding system. (**b**) Treatment with Aβ 25-35 and Aβ 1-42 at 24 hours after training both lead to memory loss 24 hours later (no feeding response to CS). Aβ 25-35, but not Aβ 1-42, targets the CGC soma and resets its membrane potential to the untrained level resulting in the removal of downstream effects of the depolarized soma membrane, which prevents the conditioned feeding response to be activated by the CS. Aβ 1-42 does not affect the CGC soma and may exert its memory impairing effect by affecting the SN to CGC, CGC to SN and/or SN to CBI synapses (areas in blue circles), which also may be affected by Aβ 25-35 in addition to its primary effect on somal membrane potential. Neither peptide affects the US pathway, the CBIs, the feeding CPG, or motoneurons and the synapses connecting these components of the feeding network and therefore the animals remain capable of responding to unconditioned food stimuli.

The proposed circuit level actions of Aβ 25-35 and Aβ 1-42 in classically conditioned animals are summarized in Fig. 8. After classical conditioning and in the absence of elevated Aβ levels, the depolarized CGC gates in the CS-mediated activation of the feeding response, while the US-mediated activation of the same circuit is mediated by a parallel pathway not affected by classical conditioning (Fig. 8a). Aβ 25-35, but not Aβ 1-42, targets the CGC and reverses its depolarized state (Fig. 8b), resulting in the loss of the feeding response to the CS. Although Aβ 1-42 also causes the loss of the conditioned response, it is not doing so by affecting the CGC soma. However, based on the detailed information we have about the feeding circuit and the findings from the present study, we can formulate some hypotheses about its cellular targets. It is unlikely to target the soma of a so far unidentified neuron that plays a key role in long-term memory similar to the CGC, as previous work has shown that the learning-induced depolarization of the CGC is sufficient for LTM^21^,^22^. Also, because the feeding Central Pattern Generator (CPG) itself remains functional, as demonstrated by the ability of the Aβ treated animals to respond to the unconditioned food stimulus (Supplementary Fig. 2a), Aβ 1-42 must be targeting a component of the CS to CPG pathway. It is unlikely to hyperpolarize the soma of the Cerebral Buccal Interneuron (CBI) type cells as previous experiments have shown that this leads to changes in the fictive feeding motor pattern^29^ that also would be manifested at the behavioral level. As the CBIs have a powerful modulatory control over the feeding CPG during sucrose-induced fictive feeding in semi-intact preparations^29^ it is also unlikely that the CBI to CPG synapses are affected by Aβ 1-42 as again, this would be manifested at the level of unconditioned feeding behavior. The most likely scenario is that Aβ 1-42 is targeting the CGC to CS sensory neuron and/or CS sensory neuron to CBI synapses (Fig. 8b) as this would only impair the animals’ response to the CS but not to the US. We cannot rule out either that it targets the CS sensory neuron to CGC input, which would reduce the CS induced weak increase in CGC spike frequency which operates in conjunction with the somal depolarization to activate the CS sensory neuron to CBI synaptic input^23^. There are numerous examples of Aβ 1-42 interfering with synaptic mechanisms mediating aspects of learning and memory in other systems^2^ so it is plausible that it acts in a similar manner in the *Lymnaea* brain. It is also possible that Aβ 25-35 has an effect on the same synapses as Aβ 1-42, however, based on the current and previous studies^21^,^22^, its effect on the CGC soma alone would be sufficient to lead to behavioral memory impairment. The Aβ induced memory impairment seems to be dependent upon the animals having been trained, since treatment prior to training caused neither memory deficits nor Aβ 25-35 induced changes in membrane properties. Further work will need to be undertaken to establish the molecular mechanisms underlying the selective effect of Aβ on cellular and molecular pathways of established memory.

## Methods

### Experimental animals

Pond snails, *Lymnaea stagnalis*, were bred at the University of Sussex and maintained in large holding tanks containing 18-22°C copper-free water, at a 12:12 hour light-dark cycle. The animals were fed Tetra-Phyll (TETRA Werke) twice a week and lettuce three times a week.

### Preparation and systemic application of Aβ peptides

Aβ 1-42 was prepared in normal saline solution^17^ for maximally soluble oligomeric morphology, as previously described^30^. Briefly, 0.2 mg Aβ 1-42 (rPeptide) was solubilized in 200 μL HFIP (Sigma-Aldrich) to disaggregate the peptide. The solution was then vortexed on high for one minute and sonicated in a 50/60 Hz bath sonicator for one minute. The HFIP was then dried completely using a low stream of nitrogen gas for five to ten minutes. To ensure complete removal of HFIP, the sample was placed in a dessicator for 30 minutes. Once completely dried, 200 μL dry DMSO (Sigma-Aldrich) was added to the Aβ 1-42, vortexed for one minute, and sonicated for one minute. The Aβ 1-42 was then added to a prepared Zeba buffer-exchange column with 40 μL normal saline solution as a stacking buffer and centrifuged for 30 minutes at 16k RPM. Using a molar absorption coefficient of 1490 M^−1^ cm^−1^, protein concentration was assessed by measuring at 280 nm using a NanoDrop spectrophotometer. The peptide was then diluted using normal saline to a final concentration of 1 μM.

To prepare Aβ 25-35 for systemic injection, 0.25 mg Aβ Fragment 25-35 (Sigma- Aldrich) was mixed with 1.25 mL copper-free water and left to incubate for two hours to allow the peptide to solubilize and assemble. After two hours, the sample was diluted with normal saline solution to a final concentration of 1 μM or 0.1 mM. The peptides were administered to the animals directly after preparation. Using a 1 mL syringe with 30 gauge precision glide needles (Becton Dickinson), 100 μL of the Aβ 25-35 or Aβ 1-42 peptide solution was injected into the hemolymph (~1 mL in volume) of each snail. The estimated final concentration in the animal was 0.1 μM for Aβ 1-42 and 0.1 μM or 10 μM for Aβ 25-35. As there is no blood-brain barrier in *Lymnaea*^17^, the injected peptides have direct access to the animal’s central nervous system. For vehicle-injected control animals, 100 μL of normal saline was injected.

### Preparation and imaging of Alexa Fluor 488 tagged Aβ 1-42

The protocol for preparation was fully described previously^30^. Briefly, an Alexa Fluor 488 Protein Labeling Kit (Invitrogen) was used to label the freshly solubilized Aβ 1-42 (described above), following the manufacturer’s instructions. Aβ 1-42 was prepared as normal up to the DMSO stage, where kit components were added to the vial and allowed to incubate for 15 minutes at 21 °C. Tagged-peptide was then run on a 2 mL Zeba buffer-exchange spin column with 40 μL normal saline solution as a stacking buffer and centrifuged for 30 minutes at 16k RPM, to remove any free tag from the solution. Protein concentration was measured as described for Aβ 1-42 preparation with the addition of a measurement at 495 nm and 0.11 correction factor to account for the added Alexa Fluor 488 tag, and degree of labeling was then assessed using the molar extinction coefficient of the Alexa Fluor 488 of 71,000 M^−1^ cm^−1^. Protein labeling was low, 0.042 moles dye per moles protein, likely due to the low concentration of peptide (less than 2 mg/mL) used, as suggested by the manufacturer. Tagged-Aβ 1-42 was diluted to 1 μM in normal saline solution.

For wholemount fluorescent imaging of Alexa Fluor 488 tagged Aβ 1-42, snail brains were dissected and the desheathed brains were immediately placed on glass slides with coverslips and imaged using an Olympus BX61WI with a 10 × 1.0 NA dipping objective and excitation and emission filters 470/22. Images were taken using an EMCCD camera (Andor iXon), processed using μManager software^31^, and binned at 1 × 1.

For TEM, buccal and cerebral ganglia were dissected and prepared for immunogold labeling using a previously described protocol involving minimal, cold fixation and embedding in Unicryl resin^30^,^32^. Briefly, thin (100 nm) sections were labeled with 1 μg/mL rabbit antibody specific for Alexa Fluor 488 (Molecular Probes). They were then labeled with a goat anti-rabbit 10 nm gold-conjugated secondary antibody (BBI Solutions OEM Ltd., Cardiff, UK) and stained with 2% aqueous uranyl acetate. The labeled thin sections were examined in a Hitachi 7100 TEM at 100 kV and digital images acquired with an axially mounted (2K x 2K pixel) Gatan Ultrascan 1000 CCD camera (Gatan UK, Oxford, UK).

### Formic acid extracted hemolymph preparation

After 24 hours *in vivo* incubation of either Aβ 1-42 or Aβ 25-35, a 1 mL syringe with 30 gauge precision glide needles (Becton Dickinson) was used to extract roughly 1 mL of hemolymph from each snail. Each sample was submitted to formic acid extraction, a method of removing soluble Aβ from tissue samples^33^. 1 mL of pooled hemolymph sample was mixed with 1 mL 0.4% diethylamine/100 mM NaCl. 400 μL was then centrifuged at 14K RPM for 1 hour at 4 °C. The supernatant was removed and 200 μL 1 M Tris Base pH 7.4 was added to the pellet. 400 μL cold formic acid was then added. The sample was sonicated for 20 seconds and then 400 μL was centrifuged at 14K RPM for 1 hour at 4 °C. 210 μL of the supernatant was diluted into 4 mL Formic Acid Neutralization Buffer (1 M Tris Base, 0.5 M Na2HPO4) and 2 mL of this mixture was centrifuged at 14K RPM for 1 hour at 4 °C. The supernatant was then neutralized with 1/10 volume 1 M Tris Base (pH 6.8). The samples were stored at −80 °C until used for imaging.

### TEM negative stain

Formic acid extracted hemolymph samples were prepared for TEM negative stain to quantify soluble, oligomeric Aβ within the animals’ body fluids. 4 μL of each sample were pipetted on to Formvar/carbon coated 400-mesh copper grids (Agar Scientific, Essex, UK) for 1 minute. Excess liquid was removed with Whatman paper. Grids were washed with 4 μL of Milli-Q water and blotted, followed by 4 μL of filtered 2% (w/v) uranyl acetate for 1 minute and blotted again. Grids were allowed to air dry before being examined in a Hitachi 7100 TEM at 100 kV and digital images acquired with an axially mounted (2K x 2K pixel) Gatan Ultrascan 1000 CCD camera (Gatan UK, Oxford, UK). After initial imaging, the samples were immunogold labeled (as previously described) to determine oligomeric structure. A 1 μg/mL mouse Nu1 primary antibody (Klein laboratory)^16^ and a goat anti-mouse 10 nm gold-conjugated secondary antibody (BBI Solutions OEM Ltd., Cardiff, UK) were used and grids were imaged as stated previously.

Negative staining of Aβ 1-42 and Aβ 25-35 was used to determine peptide morphology. Aliquots of 94 μM Aβ 25-35 and 100 μM Aβ 1-42 were allowed to incubate in normal saline solution for 0, 3, or 24 hours. Samples were prepared and images acquired as stated above.

### Single-trial food-reward classical (CS + US) conditioning

Using well-established methods^21^, four- to six-month-old snails were removed from their home tanks and starved in new tanks for two days at the same temperature and light dark cycle as the home tanks. After the starvation period, the animals underwent single-trial food-reward classical conditioning in which the CS (amyl acetate: 0.004% final concentration) and the US (sucrose: 0.6% final concentration) were paired. Initially, each individual snail was left to acclimatize in a 14 cm diameter Petri dish with 90 mL of 18-22°C copper-free water for ten minutes. After the acclimatization period, 5 mL of amyl acetate was added to the dish and after thirty seconds, 5 mL of sucrose was added. The snails were then left in their Petri dishes for two minutes, and then removed to their starvation tanks. Both the vehicle-injected and Aβ-injected groups were trained. The naïve groups were not trained, but underwent the same starvation/feeding schedule and handling.

Forty-eight hours after training, all animals were tested for LTM with the CS. Each individual snail was left to acclimatize in a 14 cm-diameter Petri dish with 90 mL of 18-22°C copper-free water for ten minutes. After the acclimatization period, 5 mL of 18-22°C copper-free water was added to the dish. Rasps, the animals’ feeding movements, were manually counted for two minutes to determine a baseline rasping rate (number of rasps per two minutes) for each individual. After two minutes, 5 mL amyl acetate was added to the dish. Rasping was tracked for two minutes. Rasping rates were determined by subtracting the individual animal’s baseline rasp from the amyl acetate induced rasp. A separate group of Aβ-treated naïve animals were tested for their ability to produce the unconditioned feeding motor response to the US 24 hours post injection. Snails from the same home tanks as those used in the classical conditioning paradigm were submitted to the same starvation and acclimatization procedures. However, these animals did not undergo training, only Aβ or vehicle injection and testing. After the acclimatization period, 5 mL of 18-22 °C copper-free water was added to the dish. Rasping was tracked for two minutes to determine a baseline rasping rate for each individual then 5 mL sucrose was added to the dish and rasping was tracked for another two minutes.

### Cell death and apoptosis measurements

For the cell morphology analysis, TEM was used to examine buccal and cerebral ganglia as detailed above. Images were then qualitatively scored for the health of the nucleolus, nuclear envelope, cellular membrane, and chromatin on a scale of 0 to 2, with 0 = unhealthy 1 = uncertain 2 = healthy.

For the Annexin V-Alexa Fluor 488 analysis, buccal and cerebral ganglia were dissected and pinned on to a sylgard square. Samples were immediately placed in 4% paraformaldehyde (in 0.2 M phosphate buffer, pH 7.2) for 1 hour and then transferred to a 30% sucrose in phosphate buffered saline (PBS) solution overnight at 4 °C. Samples were removed from the sylgard square, coated in OCT embedding medium (VWR), and frozen in liquid nitrogen. Samples were sliced to 14 μm thickness using a Cryostat and placed on to SuperFrost Plus slides (VWR). Slides were then allowed to dry for at least 30 minutes before being washed three times for two minutes each in PBS. 500 μL of 4% normal goat serum in PBS was then added to each slide for 1 hour. Slides were again washed three times for two minutes in PBS and then labeled with 100 μL Annexin V- Alexa Fluor 488 (1/500 dilution in PBS) (Invitrogen) per slide, coverslipped, and allowed to incubate in the dark at 4 °C. Slides received three final washes in PBS for two minutes each before 6 drops of Fluoroshield (Sigma Aldrich) was added to each slide, along with a coverslip. Slides were allowed to dry and samples were imaged using an Olympus BX61WI with a 10 × 1.0 NA dipping objective and excitation and emission filters 470/22. Images were taken using an EMCCD camera (Andor iXon), processed using μManager software^30^, and binned at 2 × 2. Images were analyzed using ImageJ software and measured for area, mean, integrated density, and background signal mean all on the same ImageJ default setting for threshold. Final signal intensity was calculated using the Corrected Total Ganglia Fluorescence (CTGF) formula: CTGF = Integrated Density – (Area of selected ganglia X Mean fluorescence of background reading).

The TUNEL assay (Roche) analysis was performed using the same method as the Annexin V-Alexa Fluor 488 analysis. Samples were prepared, sectioned, and added to slides as previously mentioned. Sections were permeabilized in 0.1% Triton-X in PBS for 2 minutes on ice. Sections on the positive control slides were then treated with DNase I in Tris-HCl for 10 minutes, and all slides were labeled with TUNEL reaction mixture for 60 minutes at 37 °C except for the negative control, which were only labeled with the Label Solution. Slides received two final washes in PBS for two minutes each before 6 drops of Fluoroshield (Sigma Aldrich) was added to each slide, along with a coverslip. Slides were then dried, imaged, and analyzed as previously mentioned.

### Electrophysiology

The two electrode current-clamp-based electrophysiological methods used in this study on the CGC have been described elsewhere^21^. Briefly, the CNS was dissected and pinned in a sylgard-coated dish containing *Lymnaea* physiological saline. The cerebral ganglia (location of the CGCs) were desheathed using fine forceps and treated with a solid protease (Sigma, XIV) for 1 min to soften the inner-sheath. Intracellular recordings were made using sharp electrodes (5-20 MΩ) filled with 4 M potassium acetate. Axoclamp 2A (Axon Instrument, Molecular Device) and NL 102 (Digitimer Ltd) amplifiers were used and data acquired using a micro 1401 Mk II interface and analyzed using Spike 2 software (Cambridge Electronic Design). Two-electrode current clamp techniques were used to measure the membrane properties of the CGC. The CGC membrane potential and action potential characteristics (action potential amplitude, half-width, and after-hyperpolarization amplitude) were determined over a 100 s period recorded 120 s after electrode impalement. To test CGC membrane resistance, the cell was held at −70 mV and injected with five −2 nA 2 s duration current pulses.

### Statistical analysis

Data that passed the D’Agostino and Pearson omnibus normality test were subjected to parametric tests (one-way ANOVA with Tukey’s multiple comparison, or t-tests) to establish significance (criterion, p < 0.05). GraphPad Prism software was used for all analyses.

## Additional Information

**How to cite this article**: Ford, L. *et al.* Effects of Aß exposure on long-term associative memory and its neuronal mechanisms in a defined neuronal network. *Sci. Rep.* **5**, 10614; doi: 10.1038/srep10614 (2015).

## Supplementary Material

Supporting Information
