# Supplementary material for: Effects of Aβ exposure on long-term associative memory and its neuronal mechanisms in a defined neuronal network
Source: Sci Rep. 2015 May 29;5:10614. doi: 10.1038/srep10614 (PMC4448550; doi:10.1038/srep10614)
Supplement: Supporting Information [file srep10614-s1.doc]

**Effects of A**β **exposure on long-term associative memory and its neuronal mechanisms in a defined neuronal network**

Lenzie Ford, Michael Crossley, Thomas Williams^+^, Julian R. Thorpe, Louise C. Serpell*, György Kemenes*

Sussex Neuroscience, School of Life Sciences, University of Sussex, Brighton, BN1 9QG.

^+^Present address: Case Western School of Medicine, Department of Physiology and

Biophysics, 10900 Euclid Ave, Cleveland, 44106-4970, USA

*Joint corresponding authors: G.Kemenes@sussex.ac.uk, L.C.Serpell@sussex.ac.uk


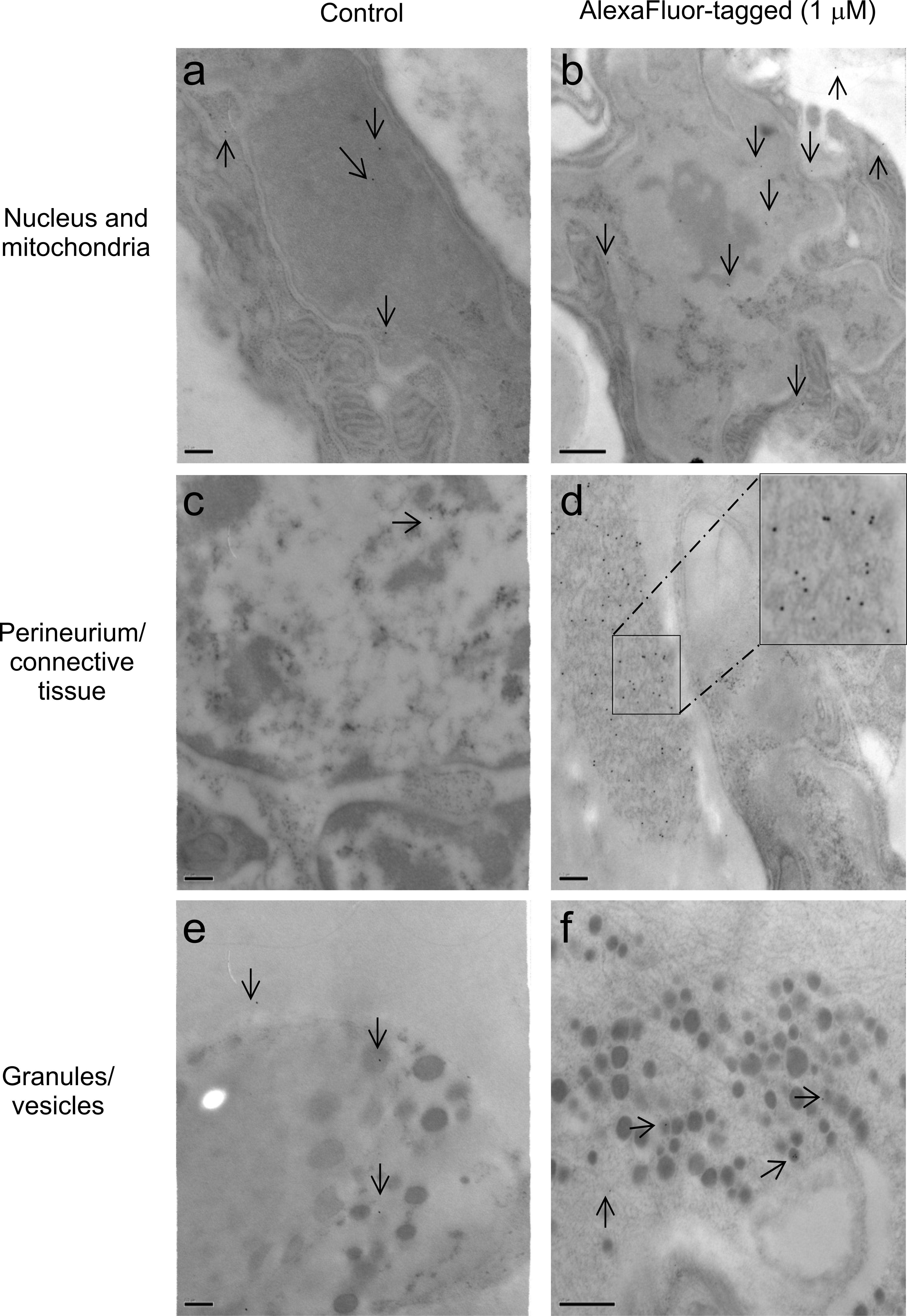
**Supplementary figures**

**Supplementary Figure 1, related to Figure 1. Alexa Fluor 488-tagged Aβ 1-42 enters the snail brain by 24 hour *in vivo* incubation at 1 μM concentration.** Sections of treated buccal ganglia were labeled with an anti-Alexa Fluor 488 primary antibody and a 10 nm gold-conjugated secondary antibody, and imaged using a transmission electron microscope (TEM). **a, b**) Areas of gold labeling are indicated with arrows. Scale bars represent 0.5 μm. **c, d**) High labeling occurs within the perineurium/connective tissue of Aβ injected animals only. Inserts are added to show areas of high labeling. Scale bars represent 0.5 μm. **e, f)** Labeling occurs in dense core granules/vesicles with Aβ application. Arrows indicate labeling and inserts are used to magnify highly labeled areas. The scale bar in image **e** represents 0.2 μm,
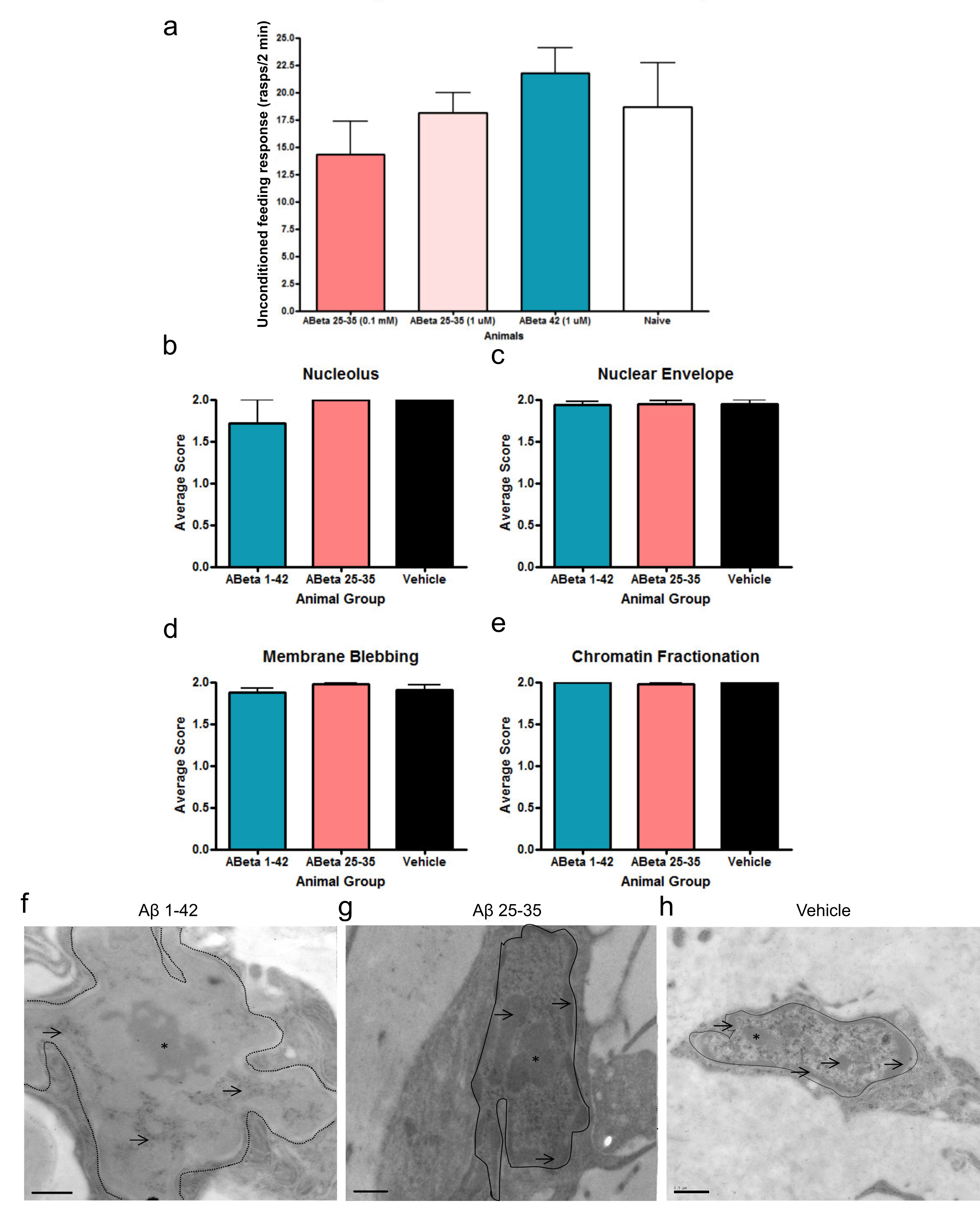
the scale bar in image **f** represents 0.5 μm.

**Supplementary Figure 2, related to Figures 3, 4 and 5. Aβ-treated animals exhibit no significant cell death characteristics, as measured by circuitry and cellular health indicators.**

**a)** Four animal groups were tested for their feeding response to the unconditioned stimulus, sucrose. Aβ 25-35 (0.1 mM) n=9, Aβ 25-35 (1 μM) n=15, Aβ 1-42 (1 μM) n=12, Naïve n=10. Means ± SEM values are shown. One-way ANOVA, p=0.3542. All Tukey’s tests: p>0.05.

**b-e**) TEM images of buccal ganglia for Aβ 1-42, Aβ 25-35, or vehicle-treated animals were qualitatively scored on a range of 0-2 (where 0= unhealthy, 1= uncertain, 2= healthy) for health of the cell based on four traits: nucleolus **b**, nuclear envelope **c**, membrane **d**, and chromatin **e**; and quantified. There was no significant change in any of the characteristics scored. **b**) Nucleolus, Aβ 1-42 n=7, Aβ 25-35 n=8, vehicle n=2. **c**) Nuclear envelope, Aβ 1-42 n=31, Aβ 25-35 n=38, vehicle n=20. **d**) Membrane blebbing, Aβ 1-42 n=47, Aβ 25-35 n=85, vehicle n=21. **e**) Chromatin fractionation, Aβ 1-42 n=29, Aβ 25-35 n=36, vehicle n=20. Means ± SEM values are shown. One-way ANOVA, p=0.3542. All Tukey’s tests: p>0.05.

**f-h**) Example TEM images of buccal ganglia. Note the integrity of the nuclear envelope (dotted line), state of chromatin (asterisk), and health of the nucleolus (arrow). The scale bar for Aβ 1-42 represents 1 μm, for Aβ 25-35 and Vehicle scale bars represent 0.5 μm.

**
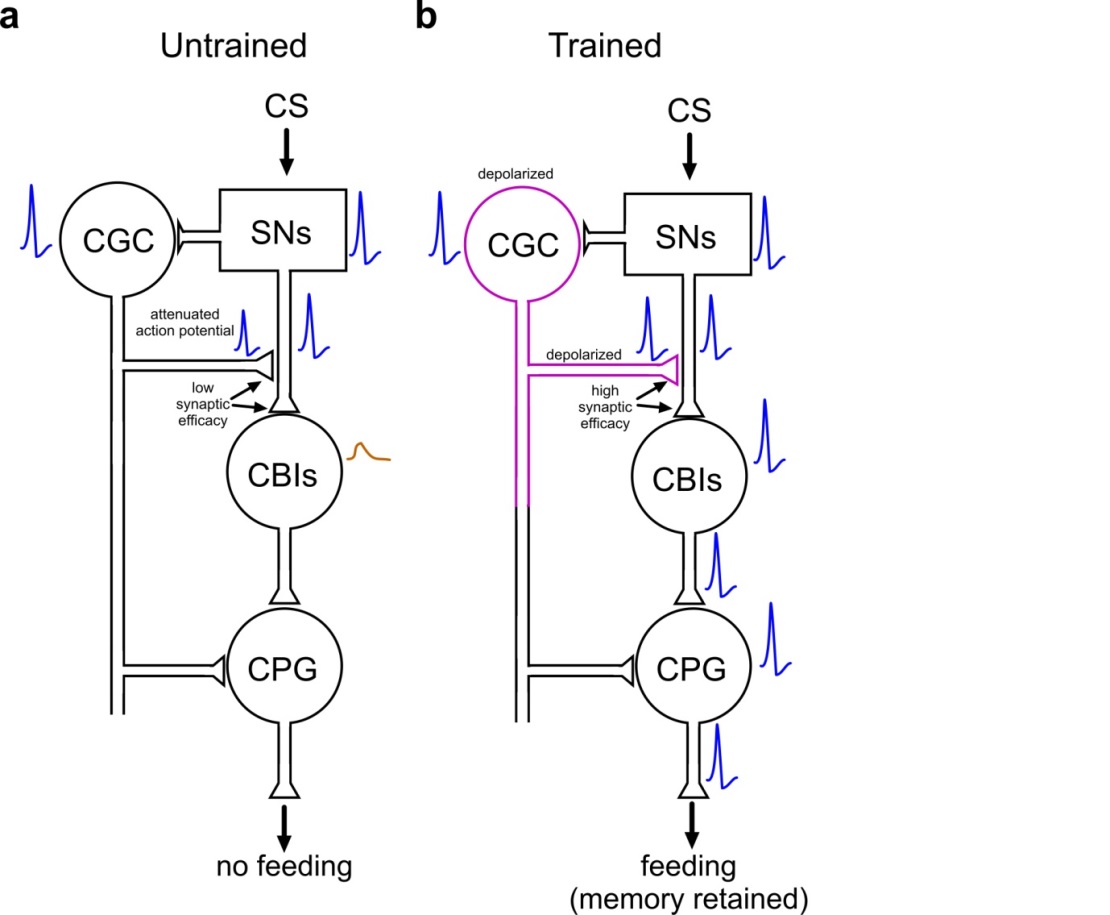
**

**Supplementary Figure 3, related to Figure 6. The cellular and circuit mechanisms of 'remote controlled' increase in synaptic efficacy after classical conditioning in *Lymnaea* (based on^1-2^).**

**a**) In untrained animals, action potentials (blue cartoon symbols) recorded in the Cerebral Giant Cell (CGC) soma strongly attenuate by the time they reach the presynaptic terminals of the neuron within the cerebral ganglia. Action potentials triggered by the conditioned stimulus (CS) in chemosensory neurons (SNs) of the lips only evoke small EPSPs (brown cartoon symbol) in the command-like Cerebral to Buccal Interneurons (CBIs) of the feeding system (low synaptic efficacy).

**b**) In conditioned animals, 24 hours after training, the membrane potential of the CGC soma and proximal axonal segments are persistently depolarized (indicated by purple outlines) resulting in a much smaller attenuation of action potentials between soma and presynaptic axon terminal compared to untrained animals. Due to a combination of increased background calcium levels and increased calcium influx during action potentials in the CGC axon, CGCs in trained animals presynaptically facilitate the output from SNs (high synaptic efficacy), resulting in action potential firing in the CBIs. The CBIs in turn will activate interneurons of the feeding central pattern generator (CPG) to generate the rhythmic firing pattern underlying the conditioned feeding response to the CS (memory retained). The learning-induced depolarization does not spread as far as the CGC to CPG synaptic connections and therefore the modulatory effect of the CGC on unconditioned feeding remains
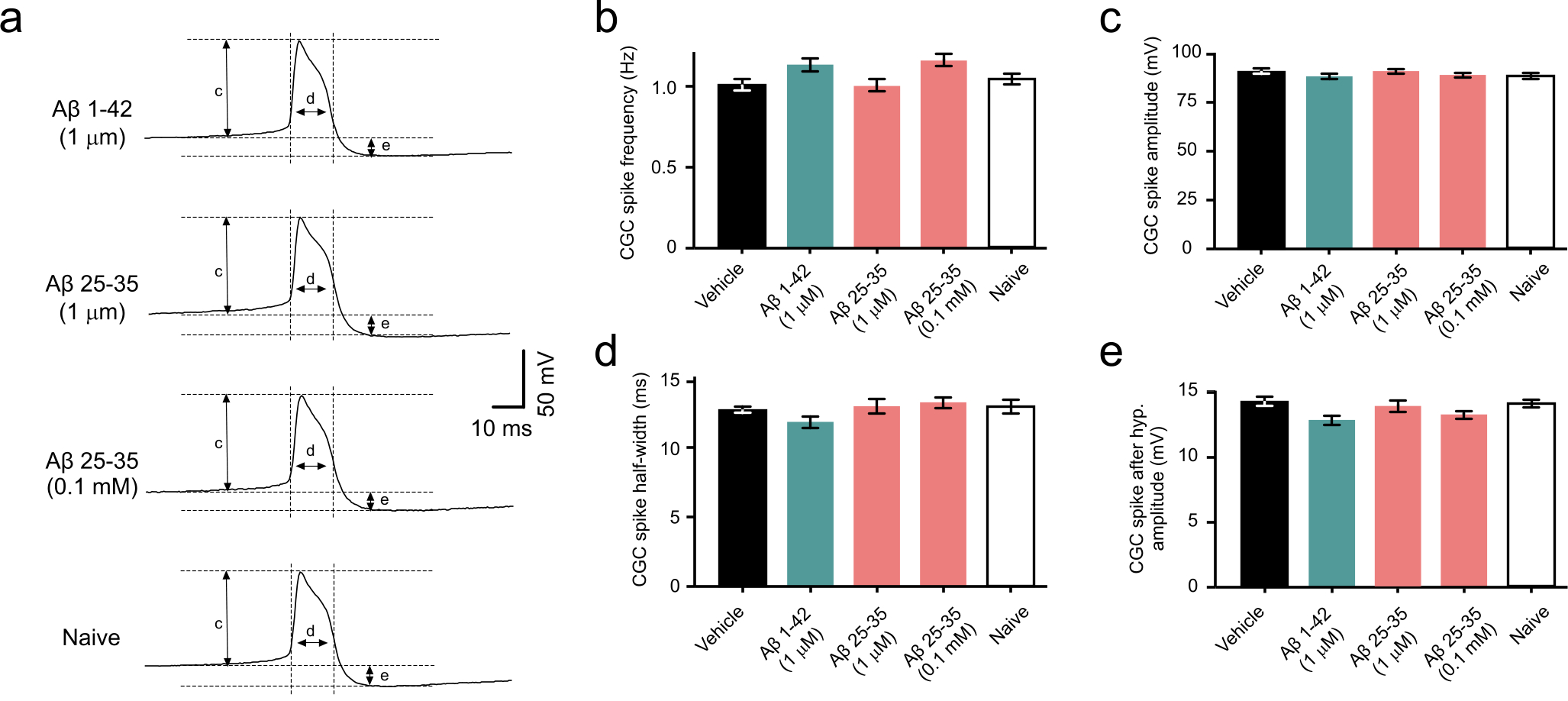
unaffected.

**Supplementary Figure 4, related to Figure 6. Aβ 1-42 and Aβ 25-35 do not alter the spike parameters of the CGC.**

**a**) Examples of electrophysiological recordings of single CGC spikes from Naïve, Vehicle-treated, Aβ 1-42-treated (1 μM), Aβ 25-35-treated (1 μM), and Aβ 25-35-treated (0.1 mM) animals.


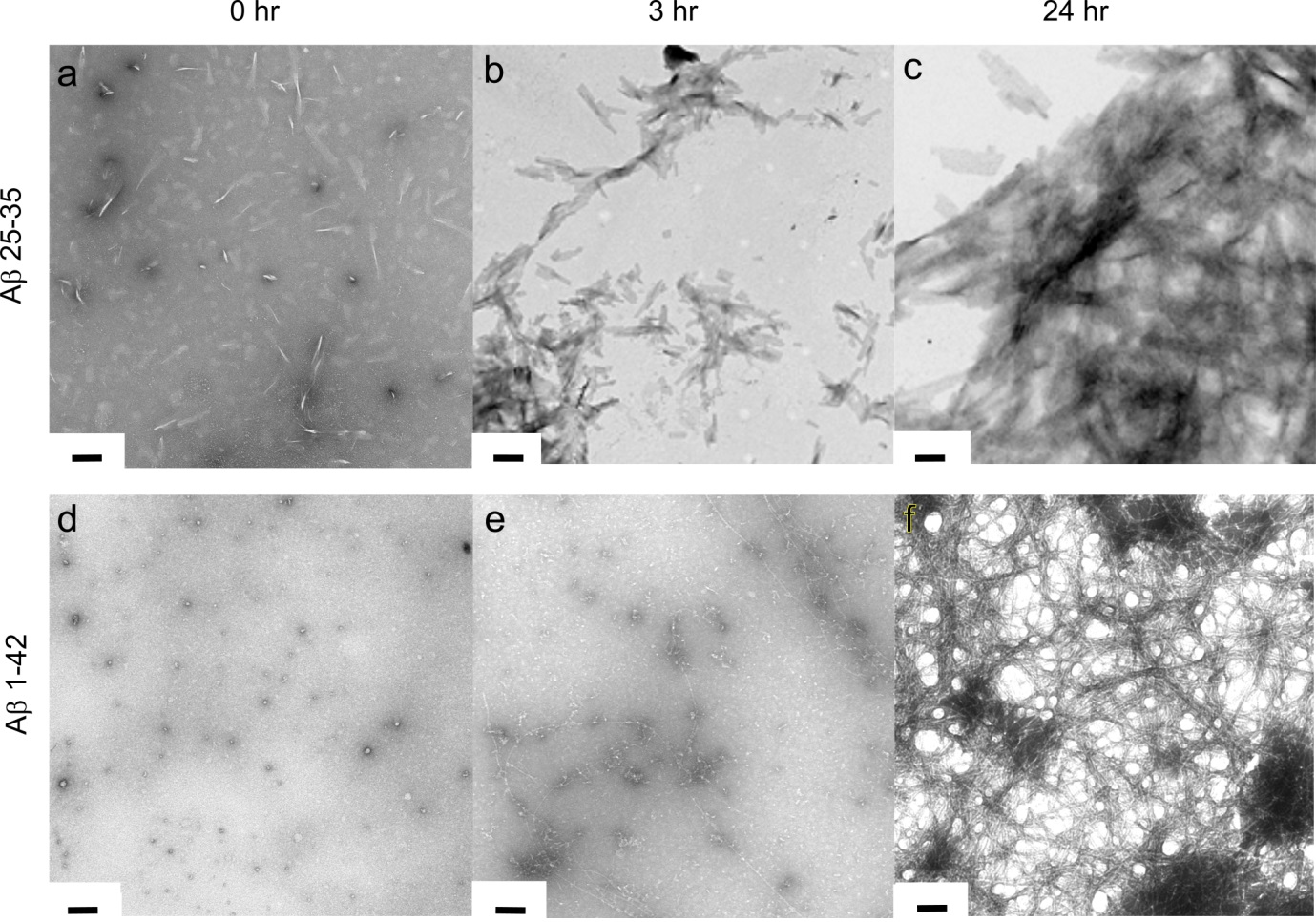
**b-e**) The analysis of four important parameters of the CGC spikes: frequency **b**, amplitude **c**, half-width **d**, and after hyperpolarization amplitude **e**. No significant effects of Aβ injection after 24 hours treatment is found. Naïve, n = 14; Vehicle, n = 13; Aβ 1-42, n =12; Aβ 25-35 (0.1 mM), n = 15; Aβ 25-35 (1 μM), n=10.

**Supplementary Figure 5, related to Results section ‘24 hour *in vivo* incubation with Aβ alters neuronal properties’. Aβ 1-42 and Aβ 25-35 aggregates differently when allowed to incubate in normal saline solution for 24 hours**

Aβ 25-35 (94 μM) and Aβ 1-42 (100 μM) were prepared as described in the Methods and allowed to aggregate in normal saline solution over a 24-hour period. Samples were taken at 0, 3, and 24 hours post-prepatation, negative stained, and imaged using TEM. Both Aβ 1-42 and Aβ 25-35 self-assemble over the 24-hour period. However, they appear to be morphologically different at each time point, whereby Aβ 25-35 forms small, wide crystalline structures almost immediately (**a-c)**, whilst Aβ 1-42 forms the expected small spherical oligomers, protofibrils and finally, mature amyloid fibrils (**d-**
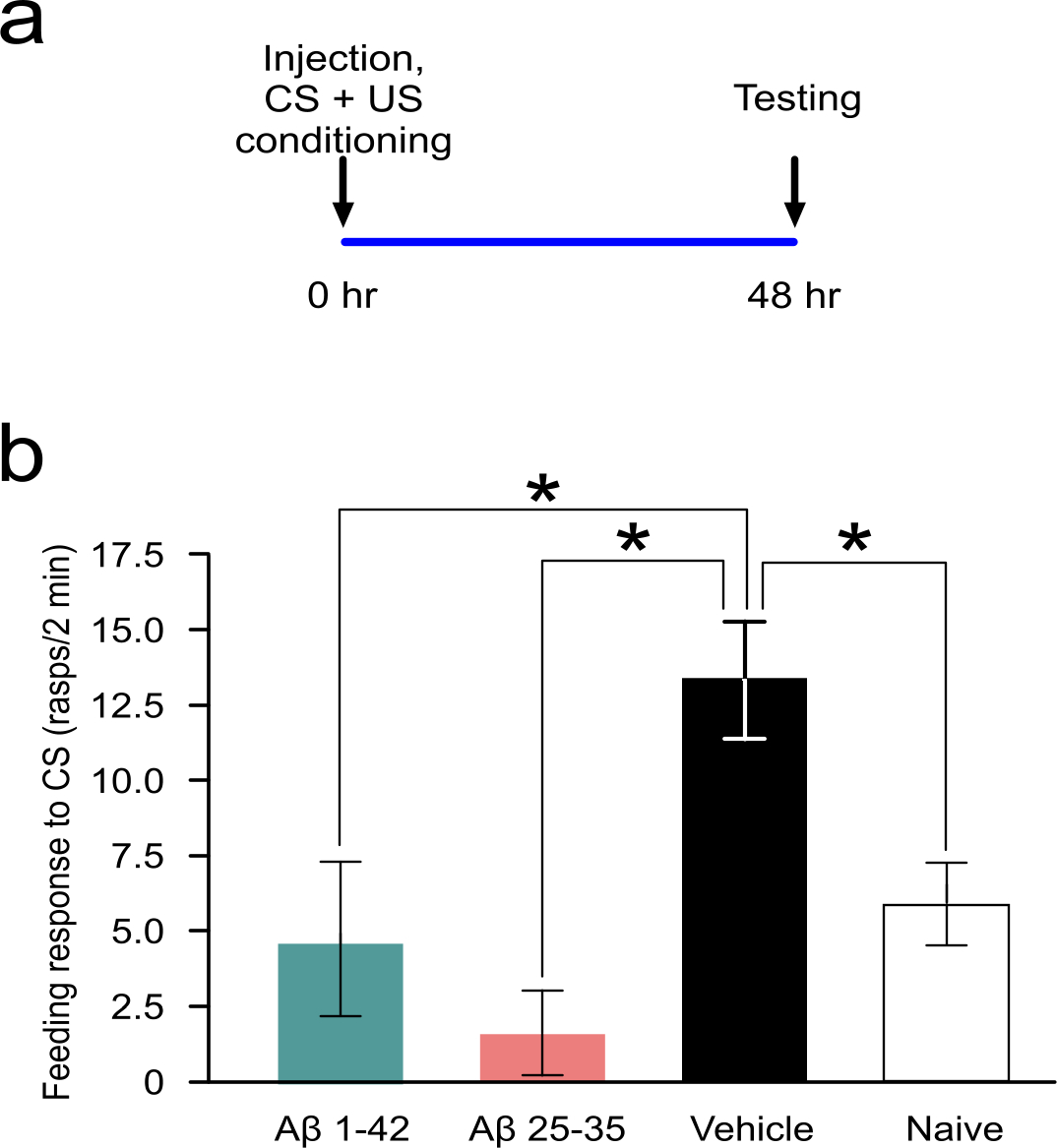
**f)**. Scale bars represent 100 nm.

**Supplementary Figure 6, related to Figure 7. Treatment with A**β **at the time of training impairs memory 48 hours later.**

**a**) Timeline of the experiment. Four starved animal groups (Aβ 1-42, n=18; Aβ 25-35, n=16; Vehicle, n=32; Naïve, n=36) were tested for the feeding response to CS 48 hours after injection and training.

**b**) Means ± SEM values for the CS-evoked feeding response are shown. Asterisks indicate responses that are significantly lower than that of the vehicle-injected group. One-way ANOVA, p=0.0002. Tukey’s tests with p<0.05: Aβ 1-42 vs Vehicle and Vehicle vs Naive. Tukey’s tests with p<0.001: Aβ 25-35 vs Vehicle.

**Supplementary references**

1 Kemenes, I. *et al.* Role of delayed nonsynaptic neuronal plasticity in long-term associative memory. *Current Biology* **16**, 1269-1279, (2006).

2 Nikitin, E. S., Balaban, P. M. & Kemenes, G. Nonsynaptic plasticity underlies a compartmentalized increase in synaptic efficacy after classical conditioning. *Current Biology* **23**, 614-619, (2013).
